# A Novel Mutation in the *AVPR2* Gene Causing Congenital Nephrogenic Diabetes Insipidus

**DOI:** 10.4274/jcrpe.0097

**Published:** 2018-11-29

**Authors:** Aslı Çelebi Tayfur, Tuğçe Karaduman, Merve Özcan Türkmen, Dilara Şahin, Aysun Çaltık Yılmaz, Bahar Büyükkaragöz, Ayşe Derya Buluş, Hatice Mergen

**Affiliations:** + These authors contributed equally to this work.; 1 Keçiören Training and Research Hospital, Clinic of Pediatric Nephrology, Ankara, Turkey; 2 Hacettepe University Faculty of Science, Department of Biology, Ankara, Turkey; 3 Keçiören Training and Research Hospital, Clinic of Pediatric Endocrinology, Ankara, Turkey

**Keywords:** AVPR2, congenital nephrogenic diabetes insipidus, mutation

## Abstract

**Objective:**

Congenital nephrogenic diabetes insipidus (CNDI) is a rare inherited disorder characterized by a renal insensitivity to arginine vasopressin (AVP). In the majority of the cases, CNDI is caused by mutations in the arginine vasopressin receptor 2 (*AVPR2*) gene. Our objective is to report a novel mutation in the *AVPR2* gene causing CNDI in a 6-year-old boy, presenting with growth failure and dull normal cognitive functions.

**Methods:**

The proband was the third off-spring of non-consanguineous parents and had polyuria (4.3 L/day), polydipsia (5 L/day). The diagnosis of CNDI was established by a water-deprivation test and a desmopressin challenge test. Genetic studies were also carried out in the mother, siblings and affected family members, since excessive fluid intake and diuresis were also reported in these individuals. All exons of the *AVPR2* gene for all participants were amplified and sequenced. Bioinformatics analysis for wild-type and mutant *AVPR2* were obtained with Swiss-Model and UCSF Chimera 1.10.2.

**Results:**

A novel, hemizygous, missense mutation was identified at the position 80^th^ in exon 2 (p.H80Y) of *AVPR2* in the proband. The proband’s mother, maternal aunt and grandmother were heterozygous and his maternal uncle was hemizygous for this mutation. Bioinformatic analysis indicates this mutation would cause significant conformational changes in protein structure.

**Conclusion:**

p.H80Y mutation will cause inappropriate folding of the protein compromising water homeostasis via *AVPR2* and AVP and leading to diabetes insipidus. We suggest that future functional investigations of the H80Y mutation may provide a basis for understanding the pathophysiology of the NDI in patients with this variant.

**What is already known on this topic?**

About 90 percent of all cases of hereditary nephrogenic diabetes insipidus result from mutations in the *AVPR2* gene. To date, more than 250 mutations have been identified comprising missense, nonsense, small insertions and deletions, large deletions and complex rearrangements in *AVPR2* gene.

**What this study adds?**

In this study, we found a novel hemizygous missense mutation in the *AVPR2* gene at the position 80^th^ in exon 2 causing congenital nephrogenic diabetes insipidus in a 6-year-old boy presenting with growth failure and dull normal cognitive functions.

## Introduction

Water is vital and hydration is important for physical and mental performance. Water balance of the body is controlled through fluid intake, which is stimulated by thirst and renal excretion of water as urine (1,2,3).

Increase in plasma osmolality or decrease in blood volume leads to secretion of arginine vasopressin (AVP), also called antidiuretic hormone (ADH), from the posterior pituitary gland (4,5). AVP increases water permeability in the renal collecting ducts by activating arginine vasopressin receptor 2 (AVPR2), one of the family of G protein-coupled receptor, located on the basolateral membrane of the kidney collecting duct cells (6,7). Binding of AVP to AVPR2 activates Gs/adenylate cyclase and leads to a series of intracellular events resulting in exocytic insertion of the water channel aquaporin-2 (AQP2) from intracellular storage compartments into the luminal membrane. This results in water reabsorption from the pro-urine by the kidney tubule after an osmotic gradient (1,8,9). Any impairment in this pathway can lead to a metabolic disease called diabetes insipidus (DI) (10). DI has two major types; a deficiency of AVP causes central DI, whereas inadequate sensitivity to AVP in the kidney leads to nephrogenic DI (NDI) (11,12).

Congenital nephrogenic DI (CNDI) is an inherited form of NDI and this disorder occurs as a result of loss-of-function mutations of the *AVPR2* or *AQP2* genes. *AQP2* gene defects cause autosomal recessive or dominant NDI and are responsible for a small percentage of NDI cases. However, loss-of-function mutations in *AVPR2* lead to X-linked recessive NDI and this accounts for 90% of cases with CNDI. In addition, X-linked NDI occurs with a frequency of 4-8/1,000,000 male live births (13,14,15,16). The well-known clinical symptoms of CNDI are polydipsia, polyuria, hypernatremia and hyperchloraemia (17,18). The cause of these symptoms is the inability of the kidney to concentrate urine. This defect may be due to loss-of-function mutations of *AVPR2* (19).

In this study, we described a novel, hemizygous, missense mutation causing a conversion of the histidine residue to tyrosine in the protein sequence, at position 80^th^ in exon 2 of *AVPR2* in a 6-year-old proband with symptoms of CNDI and an X-linked recessive family pedigree. The pedigree became evident after investigating affected and unaffected family members.

## Methods

A 6-year-old boy was referred to the Pediatric Nephrology Department of Keçiören Training and Research Hospital for abnormal fluid intake (5 L/day) and excessive urine output (4.3 L/day), reported to have started in the early phases of the patient’s life. The parents indicated that it was possible to make him stop crying only if he drank water in addition to breast milk. His height and weight were less than his peers in preschool and he succeeded moderately well in preschool activities. He was the third child of non-consanguineous parents. His maternal grandmother, aunt and uncle were also reported to have excessive fluid intake and high urine output. This was the patient’s first admission to a medical center for his symptoms of polyuria and polydipsia. The values of his height, weight, blood pressure, skin turgor, serum sodium (Na), potassium, chloride, calcium, phosphorus, albumin, urea, creatinine, urine specific gravity, plasma vasopressin were obtained from physical and laboratory examinations. His serum and urinary osmolality were calculated using the ffollowing formulas, 2 x [serum Na+] + [blood urea nitrogen (BUN) ÷ 2.8] + (serum glucose ÷ 18) and 1.86 x [urine Na+] + [urea nitrogen (UN) ÷ 2.8] + (urine glucose ÷ 18) + 9. The water deprivation test and desmopressin (DDAVP) challenge test was also performed. The patient’s blood pressure and heart rate were measured. In addition, his urinary ultrasonography, pituitary magnetic resonance imaging, verbal and performance tests of the Wechsler Intelligence Scale for Children fourth edition (WISC-IV) were performed.

The collection of blood samples from the proband and his family members was approved by the Ethics Committee of the Faculty of Medicine of Hacettepe University (approval no: 2607). Written informed consent was obtained from all participants and/or their parents.

### DNA Isolation

Genomic DNA was extracted from peripheral blood leukocytes following a standard protocol using QIAamp^®^ DNA Blood Mini Kit (Qiagen, Hilden, Germany). The extracted genomic DNA was quantified spectrophotometrically (Quawell Technology, Inc., United States) and stored in aliquots at -20ºC until use.

### PCR Amplification and Direct Sequencing of *AVPR2* Gene

The entire coding regions and flanking intronic sequences of the *AVPR2* gene were amplified from genomic DNA using polymerase chain reaction (PCR). The sequences of the primers that were used in PCR are as follows: Exon 1, forward 5’-GTC TGACCA TCC CTC TCA ATC TTC-3’and reverse 5’-GGA GTC GGG AAG AGG GCC TGG TTA-3’; Exon 2a, forward 5’-ATA ACA TGG CTT CCT GGA GTC CC-3’ and reverse 5’-TGC GCT GGG CGA AGA TGA AGA GCTG-3’; Exon 2b, forward 5’-TGG AAG TGG GGC TCACTG GAA CCG GC-3’ and reverse 5’-GCT GTT GAG GCT GGC CAG CAA ACA TG-3’; Exon 3, forward 5’-TGT AGC CGT GGC TAG GGC TGA CGG G-3’ and reverse 5’ CCT GCC CCA GGA AGG CAG CTG AGC-3’. All PCR amplifications were performed under the following conditions: 45 seconds at 95 ºC, 45 seconds at 64 ºC and 45 seconds at 72 ºC for 32 cycles. After amplification, PCR products were separated by electrophoresis in 1.5% agarose gel. These products were purified enzymatically and sequenced using the Big Dye kit (Applied Biosystems, Foster City, CA, USA).

### Analyses of Bioinformatics

Three-dimensional protein structures for wild-type and mutant AVPR2 proteins, comparing amino acid sequence properties and predictions of binding sites of these proteins, were obtained with computational tools including Swiss-Model and UCSF Chimera 1.10.2 servers.

To predict the functional effect of mutation on the resulting mutant AVPR2, it was also analysed using Phenotyping Polymorphism v2 (PolyPhen-2) software.

## Results

**Figure 1:**
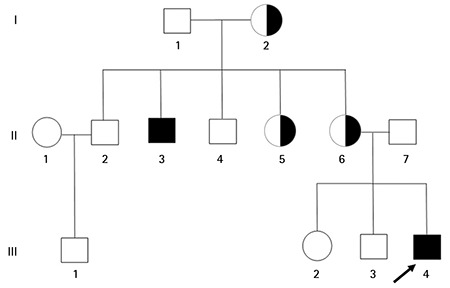
Pedigree of the family. The individuals marked with numbers are those who were available for mutation screening of the *AVPR2* gene. Black and white symbols represent clinically affected and unaffected individuals, respectively. Arrow represents proband.

At physical examination, the patient’s height [104 cm, standard deviation scores (SDS): -2.66] and weight (14 kg, SDS: -3.92) were below the 3rd percentile, his blood pressure and skin turgor were normal. Laboratory examination disclosed the following values: serum Na 137 mmol/L (range: 136-146), potassium 4.86 mmol/L (range: 3.5-5.1), chloride 102 mmol/L (range: 101-109), calcium 9.52 mg/dL (range: 8.8-10.6), phosphorus 5.67 mg/dL (range: 4-7), albumin 4.07 g/dL, urea 23 mg/dL and creatinine 0.59 mg/dL. Urine specific gravity was 1.000. The patient’s calculated serum osmolality was high 281.5 mOsm/kg, his calculated urinary osmolality was low 67.7 mOsm/kg and he had hyponatriuria (urine Na+: 15 mmol/L). The water deprivation test was stopped due to a weight loss exceeding 3% at 3.5^th^ hour of the test. Plasma vasopressin (16.75 pmol/l, range: 0-13) at the conclusion of the dehydration test was high and the urine parameters showed insignificant changes. The administration of DDAVP, 20 mg intranasally, also did not have any effect on urine parameters (Table 1). A high urine output, low urine osmolality and impaired ability to increase urine osmolality to normal levels were noted after ADH administration. The patient’s blood pressure and heart rate were stable at 112/75 mmHg and 82/min during the test. His urinary ultrasonography and pituitary magnetic resonance imaging showed no abnormality. On the verbal and performance tests of the WISC-IV, the child obtained a score of 81 and 82, respectively. Both verbal and performance tests indicated a “dull normal” intellectual function. The patient was treated with thiazide diuretics (2 mg/kg/day), indomethazine (2 mg/kg/day), low-protein diet, low Na diet and unlimited amounts of fluid. He responded well to the treatment during the first year; fluid intake (3-3.5 L/day) and diuresis (2.7-3 L/day) were both diminished. At no time during follow-up did the patient develop hydronephrosis nor did he experience dehydratation and/or hypernatremia. The family pedigree was compatible with a presumed X-linked recessive CNDI due to similar symptoms associated with NDI in some of his family members (Figure 1).

**Figure 2:**
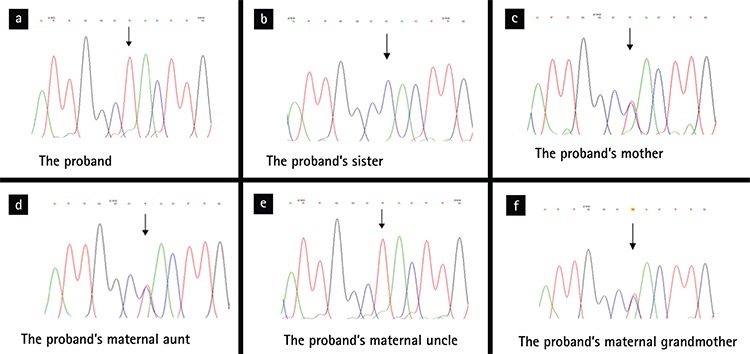
(a, b, c, d, e, f) DNA sequencing results from a part of exon 2 of the *AVPR2* gene of proband and his family members. Arrows represent the mutation site.

All of the coding regions for the *AVPR2* gene of the proband were screened by DNA sequence analysis. The analysis revealed the presence of a novel hemizygous missense mutation at coding position 238 (c.C238T) in exon 2 (Figure 2). This mutation leads to a conversion of the histidine residue to tyrosine in the protein sequence, at position 80. After the screening of the novel mutation, we also performed sequence analysis in the proband’s close family members. The results showed that his mother (II-6), maternal aunt (II-5) and grandmother (I-2) were heterozygous and maternal uncle (II-3) was hemizygous for this mutation (Figure 2). No mutation in *AVPR2* gene was detected in other family members (II-2, II-4, III-1, III-2, III-3).

**Figure 3:**
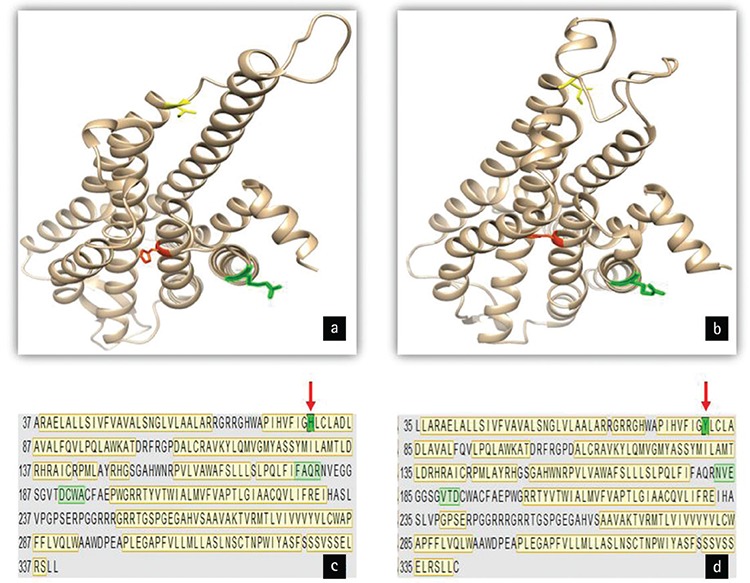
Three-dimensional protein structure predictions of wild-type (a) and mutant (b) *AVPR2*. Primary structures of wild-type (c) and mutant (d) *AVPR2* proteins. Yellow boxes represent α-helixs structures and light green boxes represent β-sheet structures. Arrows represent mutation site (processed by UCSF Chimera 1.10.2.).

According to bioinformatic analysis, based on DNA sequence, we made a prediction on mRNA structures of the wild-type and mutated proteins. H80Y mutation is located in the second transmembrane domain (amino acid positions 78-98) of the protein (20). Using the Swiss-Model and UCSF Chimera 1.10.2 servers, we found some differences between alpha-helix and beta-sheet structures of wild-type and mutant AVPR2 proteins (Figure 3). Comparisons of amino acid sequences revealed that theoretical pI values of wild type and mutant protein are 9.49 and 9.47, respectively and their theoretical molecular weights are 40,279.09 and 40,305.12, respectively. In addition, PolyPhen-2 analyses predicted the effect of the mutation with a score of 0.999 (out of 1), sensitivity of 0.14 and specificity of 0.99, therefore p.H80Y mutation was identified as a probable damaging mutation.

## Discussion

CNDI is a rare disease, most commonly caused by mutations in the AVPR2 in the collecting duct epithelial cells, which is encoded by the *AVPR2* gene (Xq28). The gene encodes a 371-amino acid, G protein-coupled receptor with seven transmembrane, four extracellular and four cytoplasmic domains (7,14). *AVPR2* mutations causing CNDI can vary in their functional severity; clinical symptoms and the response to DDAVP can be diverse.

CNDI is a severe form of DI and is difficult to treat. It is commonly due to inherited defects (14). The urine output in patients with CNDI can be lowered with a low-salt, low-protein diet, thiazide diuretics and/or potassium-sparing diuretic (amiloride) and non-steroidal anti-inflammatory drugs (21). Diuretics in NDI patients reduce theurine output by promoting the reabsorption of Na and water in the proximal tubule, thus delivering less water to the collecting ducts (22). The inhibitory effect of indomethacin on urine volume is thought to be mediated by an AVP-independent water reabsorption resulting from an increase in solute reabsorption and consequent medullary hypertonicity (23). Nevertheless, many patients still experience significant polyuria and polydipsia while receiving these therapeutic measures. The investigational therapeutic strategies for CNDI include the rescue of AVPR2 mutants by chemical chaperones and by passing defective AVPR2 signaling (24). In infants, early recognition is very important as the proper treatment can avert the physical and mental retardation that results from repeated episodes of dehydration and hypernatremia. Patients with CNDI should be monitored for growth, serum Na concentration and hydration status, and also for the development of hydronephrosis. Genetic analyses for CNDI can also be a key aid in obtaining diagnosis at an early age and should be performed in all patients with a family history of the disorder (14,21,25). Therefore, the definitive diagnosis of CNDI will provide appropriate genetic counseling and development of specific treatment strategies.

To date, more than 250 putative disease-causing *AVPR2* mutations have been found comprising missense, nonsense, small insertions and deletions, large deletions and complex rearrangements in the *AVPR2* gene (14,26). The most common category of *AVPR2* mutations causing NDI are missense mutations. Many disease-causing mutations occur in the transmembrane domains compared to extracellular or intracellular domains. *AVPR2* missense mutations are likely to impair folding and lead to rapid degradation of the misfolded polypeptide (14,25,26,27).

We report here a male child with repeated episodes of dehydration, polyuria and polydipsia in early infancy. The water deprivation test and the DDAVP challenge test confirmed the diagnosis of CNDI. The genetic analysis revealed a novel, X-linked recessive, missense mutation (p.H80Y) causing replacement of histidine residue with tyrosine in the protein sequence of AVPR2. Histidine is a basic, polar, positively charged amino acid and tyrosine is an aromatic, non-polar amino acid. The function of the altered AVPR2 structure is presumably significantly impaired as tyrosine joins in beta-strand conformations in proteins (26) in addition to the physicochemical differences between histidine and tyrosine amino acids. Therewith, an altered protein conformation might impair the intracellular trafficking of AVPR2 and affect proper localization of the receptor into plasma membrane or decrease AVP binding characteristics (20). The novel mutation reported in this study is located in the second transmembrane domain of the protein. This mutation point (codon 80) is a conserved residue among rat V1 and V2 vasopressin receptors and the human oxytocin receptor (28,29). A different missense mutation at codon 80 (p.H80R) was previously reported by Yuasa et al (20) and the authors emphasized the sequence conservation and functional importance of this codon.

The functional significance of this mutation was analysed by utilizing the PolyPhen-2 software. This software can be used to predict the consequence of an amino acid change on the structure and function of a protein using physical and evolutionary comparative considerations (18,30). As a result of the analysis, the H80Y mutation was identified as a probable pathogenic mutation. In addition, according to the bioinformatic analysis of this mutation, protein conformation is predicted to be impaired which may also lead to abnormal protein function. However, definitive functional analyses of this mutation are needed to determine the structure-function relationship in patients with CNDI.

### Study Limitation

The limitation of this study is the lack of mutation function analysis data.

## Conclusion

In conclusion, this study reports the clinical and molecular characterization of a novel mutation in *AVPR2* resulting in CNDI and emphasizes the importance of definitive diagnosis in CNDI patients.

## Figures and Tables

**Table 1:**
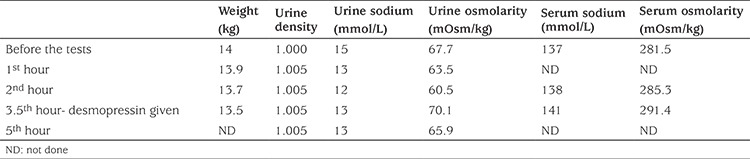
Results of the water-deprivation and desmopressin challenge tests

## References

[1] Popkin BM, D’Anci KE, Rosenberg IH (2010). Water, hydration, and health. Nutr Rev.

[2] Jequier E, Constant F (2010). Water as an essential nutrient: the physiological basis of hydration. Eur J Clin Nutr.

[3] Verbalis JG, Berl T (2007). Disorders of Water Balance. In: Brener BM. Brener and Rector’s the Kidney. Saunders.

[4] Robertson GL (1987). Physiology of ADH secretion. Kidney Int Suppl.

[5] Mavani GP, DeVita MV, Michelis MF (2015). A Review of the Nonpressor and Nonantidiuretic Actions of the Hormone Vasopressin. Front Med (Lausanne).

[6] Morello JP, Bichet DG (2001). Nephrogenic Diabetes Insipidus. Annu Rev Physiol.

[7] Juul KV, Bichet DG, Nielsen S, Norgaard JP (2014). The physiological and pathophysiological functions of renal and extrarenal vasopressin V2 receptors. Am J Physiol Renal Physiol.

[8] Chen CH, Chen WY, Liu HL, Liu TT, Tsou AP, Lin CY, Chao T, Qi Y, Hsiao KJ (2002). Identification of mutations in the arginine vasopressin receptor 2 gene causing nephrogenic diabetes insipidus in Chinese patients. J Hum Genet.

[9] Christensen JH, Rittig S (2006). Familial neurohypophyseal diabetes insipidus-an update. Semin Nephrol.

[10] Shapiro M, Weiss JP (2012). Diabetes Insipidus: A Review. J Diabetes Metab.

[11] Makaryus AN, McFarlane SI (2006). Diabetes insipidus: diagnosis and treatment of complex disease. Cleve Clin J Med.

[12] Saglar E, Deniz F, Erdem B, Karaduman T, Yönem A, Cagiltay E, Mergen H (2014). A large deletion of the AVPR2 gene causing severe nephrogenic diabetes insipidus in a Turkish family. Endocrine.

[13] Sasaki S, Chiga M, Kikuchi E, Rai T, Uchida S (2013). Hereditary nephrogenic diabetes insipidus in Japanese patients: analysis of 78 families and report of 22 new mutations in AVPR2 and AQP2. Clin Exp Nephrol.

[14] Bichet DG, Bockenhauer D (2016). Genetic forms of nephrogenic diabetes insipidus (NDI): Vasopressin receptor defect (X-linked) and aquaporin defect (autosomal recessive and dominant). Best Pract Res Clin Endocrinol Metab.

[15] Rege T, Polsani S, Jim B (2015). A rare case of conjenital diabetes insipidus. Front Med (Lausanne).

[16] Kalra S, Zargar AH, Jain SM, Sethi B, Chowdhury S, Singh AK, Thomas N, Unnikrishnan AG, Thakkar PB, Malve H (2016). Diabetes insipidus: The other diabetes. Indian J Endocr Metab.

[17] Park YJ, Baik HW, Cheong HI, Kang JH (2014). Congenital nephrogenic diabetes insipidus with a novel mutation in the aquaporin 2 gene. Biomed Rep.

[18] Guo WH, Li Q, Wei HY, Lu HY, Qu HQ, Zhu M (2016). A novel AVPR2 gene mutation of X-linked congenital nephrogenic diabetes insipidus in an Asian pedigree. J Int Med Res.

[19] Böselt I, Tramma D, Kalamitsou S, Niemeyer T, Nykänen P, Gräf KJ, Krude H, Marenzi KS, Di Candia S, Schöneberg T, Schulz A (2012). Functional characterization of novel loss-of-function mutations in the vasopressin type 2 receptor gene causing nephrogenic diabetes insipidus. Nephrol Dial Transplant.

[20] Yuasa H, Ito M, Oiso Y, Kurokawa M, Watanabe T, Oda Y, Ishizuka T, Tani N, Ito S, Shibata A (1994). Novel mutations in the V2 vasopressin receptor gene in two pedigrees with congenital nephrogenic diabetes insipidus. J Clin Endocrinol Metab.

[21] Spanakis E, Milord E, Gragnoli C (2008). AVPR2 variants and mutations in nephrogenic diabetes insipidus: review and missense mutation significance. J Cell Physiol.

[22] Wesche D, Deen PM, Knoers NV (2012). Congenital nephrogenic diabetes insipidus: the current state of affairs. Pediatr Nephrol.

[23] Dayal D, Verma Attri S, Kumar Bhalla A, Kumar R (2015). Response to low dose indomethacin in two children with nephrogenic diabetes insipidus. Pediatr Endocrinol Diabetes Metab.

[24] Milano S, Carmosino M, Gerbino A, Svelto M, Procino G (2017). Hereditary Nephrogenic Diabetes Insipidus: Pathophysiology and Possible Treatment. An Update. Int J Mol Sci.

[25] Arthus MF, Lonergan M, Crumley MJ, Naumova AK, Morin D, De Marco LA, Kaplan BS, Robertson GL, Sasaki S, Morgan K, Bichet DG, Fujiwara TM (2000). Report of 33 novel AVPR2 mutations and analysis of 117 families with X-linked nephrogenic diabetes insipidus. J Am Soc Nephrol.

[26] Kimura T, Tanizawa O, Mori K, Brownstein MJ, Okayama H (1992). Structure and expression of a human oxytocin receptor. Nature.

[27] Lolait SJ, O’Carroll AM, McBride OW, Konig M, Morel A, Brownstein MJ (1992). Cloning and characterization of a vasopressin V2 receptor and possible link to nephrogenic diabetes insipidus. Nature.

[28] Morel A, O’Carroll AM, Brownstein MJ, Lolait SJ (1992). Molecular cloning and expression of a rat V1a arginine vasopressin receptor. Nature.

[29] Bockenhauer D, Bichet DG (2015). Pathophysiology, diagnosis and management of nephrogenic diabetes insipidus. Nat Rev Nephrol.

[30] http://genetics.bwh.harvard.edu/pph2/.

